# Thyroid Autoimmunity and Antiphospholipid Syndrome: Not Such a Trivial Association

**DOI:** 10.3389/fendo.2017.00175

**Published:** 2017-07-21

**Authors:** Mathilde Versini

**Affiliations:** ^1^Department of Internal Medicine, Archet-1 Hospital, University Hospital of Nice, Nice, France

**Keywords:** antiphospholipid syndrome, autoimmunity, thyroid, Hashimoto’s thyroiditis, Graves’ disease, autoimmune disease

## Abstract

Antiphospholipid syndrome (APS) is an autoimmune disease that manifests as recurrent venous or arterial thrombosis and/or pregnancy-related complications in the presence of persistent antiphospholipid (aPL) antibodies measured at least 3 months apart. APS occurs either as a primary condition or as a part of an underlying disorder, usually systemic lupus erythematosus (SLE). Otherwise, APS may be frequently associated with autoimmune disorders. Little is known about the association of APS and aPL antibodies with thyroid autoimmune diseases or thyroid autoantibodies. This is even more interesting that thyroid autoantibodies and aPL are both recognized causes of repeated miscarriages. Therefore, their combination is of particular importance in women of childbearing age. Several studies have pointed out an association between APS and thyroid autoimmunity, some of them suggesting common pathophysiologic processes and genetic background. A literature review was conducted on existing data on aPL/APS and thyroid autoimmune disorders, paying particular attention to the possible role of this association in obstetrical complications.

## Introduction

Autoimmune thyroid diseases (AITD) encompass a spectrum of disorders characterized by a T-helper (Th)-1-cell-mediated autoimmune attack on the thyroid gland resulting in a lymphocytic infiltration of the thyroid parenchyma (1). AITD comprise two main presentations: Hashimoto’s thyroiditis (HT) and Graves’ disease (GD) corresponding to hypothyroidism and thyrotoxicosis, respectively.

The prevalence of AITD is estimated to be 5%, nevertheless the prevalence of antithyroid antibodies without clinical disorder may be even higher (1). HT (also named chronic autoimmune thyroiditis or autoimmune hypothyroidism) is the most common autoimmune disease with an incidence ranging from 27 to 448 per 100,000 per year according to the studies and the geographic areas (2), the most common endocrine disorder (3), as well as the most frequent cause of hypothyroidism (4). Its biological hallmark is the presence of antibodies directed to thyroid antigens, namely, thyroperoxydase (TPO) and thyroglobulin (Tg) (5). Similarly, GD is one of the most prevalent autoimmune diseases with an annual incidence of about 14 per 100,000 and is associated with serum antithyroid stimulating hormone receptor antibodies (6).

Associations between thyroid autoimmunity (TAI) (AITD or isolated antithyroid antibodies positivity) and other organ-specific or systemic autoimmune disorders have been widely reported. Especially, type-1 diabetes, Addison’s disease, vitiligo, alopecia, pernicious anemia, and celiac disease can be observed as part of type II or type III polyglandular autoimmune syndromes (1). Otherwise, TAI has been frequently reported in patients with systemic rheumatologic autoimmune conditions, such as systemic sclerosis (SS), Sjögren’s syndrome, rheumatoid arthritis (RA), or systemic lupus erythematosus (SLE) (7).

Antiphospholipid syndrome (APS) is an autoimmune disorder associated venous or arterial thrombosis and/or pregnancy-related complications in the presence of persistent antiphospholipid (aPL) antibodies. APS occurs either as a primary condition or as a part of an underlying disease, usually SLE. Little is known about the association between APS and TAI. This is even more important considering that both antithyroid antibodies and aPL antibodies are major causes of recurrent miscarriage (RM).

Therefore, a literature review was conducted on existing data on the association of aPL antibodies/APS with AITD/TAI, paying particular attention to the possible role of this association in obstetrical complications.

## Association Between TAI and APS

Several case reports and small case series have investigated the presence of aPL antibodies in patients with AITD and the clinical significance of such an association (8–14) (reported in Table 1). The largest cohort is reported by Tektonidou et al. (14) who found a 12% prevalence of anticardiolipin (ACL) antibodies in 168 AITD patients compared with 0% in 75 healthy controls. Paggi et al. (11) and Nabriski et al. (12) found positive titers of aPL antibodies in 43% (ACL) and 54.8% (type not specified) of AITD patients, respectively (no control groups). Interestingly in Paggi’s cohort (11), highest aPL levels were observed in GD patients with severe thyrotoxicosis and decreased following methimazole therapy. In Nabriski’s survey (12), most aPL positive patients (86%) had IgG subtype. Marongiu et al. (8) found a 38% ACL positivity in 65 GD patients vs 0% in 58 controls. In HT, Osundeko et al. (13) observed a 21% prevalence of ACL antibodies. Conversely, two other studies (9, 10) failed to show a difference between patients and healthy controls.

**Table 1:** Association between TAI and APS.

Study	Number of subjects	Population	Antibody	Outcome
Marongiu et al. (8)	65 patients	GD patients	ACL	Positive aPL in 38% of patients vs 0% of controls
	58 controls			
Petri et al. (9)	52 patients	GD (26) and HT (26) patients	ACL	No difference between patients and controls
	26 controls			
Díez et al. (10)	69 patients	AITD patients	ACL	No difference between patients and controls
	43 controls			
Paggi et al. (11)	31 patients	AITD patients	ACL	Positive aPL in 43% of patients
	No controls			
Nabriski et al. (12)	130 patients	AITD patients	aPL (type not specified)	Positive aPL in 54.8% of patients
	No controls			
Osundeko et al. (13)	19 patients	HT patients	ACL	Positive aPL in 21% of patients
	No controls			
Tektonidou et al. (14)	168 patients	AITD patients	ACL	Positive aPL in 12% of patients vs 0% of controls
	75 controls			
Innocencio et al. (26)	13 patients 163 controls	APS patients (not specified as primary or secondary)	TgAb, TPOAb	No TAI positivity in APS patients
		SS (25), RA (25), and healthy (113) controls		
Mavragani et al. (27)	75 patients 150 controls	APS patients (40 primary, 35 APS secondary to SLE)	TgAb, TPOAb	1. No significant difference between the 3 groups
				2. SLE-APS have increased TPOAb
		SLE (75) and healthy (75) controls		3. APS patients with TAI have more CNS involvement
de Carvalho and Caleiro (28)	50 patients	Primary APS patients	TgAb, TPOAb, TRAb	Positive TAI in 18% of patients
	No control			
De Carolis et al. (29)	203 patients 162 controls	Primary obstetrical APS patients	TgAb, TPOAb	Positive TAI in 27% of APS patients
		Controls with TAI and RM		

*ACL, anticardiolipin; AITD, autoimmune thyroid diseases; aPL, antiphospholipid; APS, antiphospholipid syndrome; CNS, central nervous system; GD, Graves’ disease; HT, Hashimoto’s thyroiditis; RA, rheumatoid arthritis; RM, recurrent miscarriage; SLE, systemic lupus erythematosus; SS, systemic sclerosis; TAI, thyroid autoimmunity; TgAb, antithyroglobulin antibody; TPOAb, antithyroperoxydase antibody; TRAb, thyroid receptor antibody*.

Importantly, in all of these series none of the patients with positive aPL antibodies demonstrated APS manifestations. Rare case reports (15–20) describe patients presenting with concomitant GD and APS with thrombotic manifestations. There is no reported association of HT with thrombotic manifestations of APS. It is to note that GD has been implicated as a procoagulant state since many years, through several mechanisms including elevated factor VIII and fibrinogen levels, and increased factor X activity (21–23). Therefore, it is not surprising that the association of hypercoagulability inherent to the activity of GD and the presence of aPL antibodies may conduct to thrombotic manifestations. Apart from these rare cases, to date in the view of available data, aPL positivity during the course of AITD appear to be an epiphenomena without clinical impact. Indeed, aPL antibodies are often detected in patients with autoimmune disorders but the occurrence of clinical manifestations of APS remains scare.

Thus, such antibodies are presumed to result from an excessive stimulation of B lymphocyte clones with autoreactive potential (12). In addition, Hofbauer et al. (16) hypothesized a molecular mimicry between the epitopes of TSH receptors and β2 glycoprotein 1 as a possible pathogenic mechanism. Benvenga and Guarneri (24) explored this hypothesis through an *in silico* study and found homologies between various microorganisms and thyroid antigens. Dagenais et al. (25) conducted an elegant immunogenetic study on family members with autoimmune diseases highlighting that HLA DR4 and DR7 antigens could predispose patients with GD and high titers of ACL antibodies to develop the full clinical spectrum of APS.

Few studies have investigated the relationship in the other direction, that is the prevalence of thyroid autoantibodies or AITD in APS patients (26–29) (reported in Table 1). In 2010, de Carvalho and Caleiro (28) evaluated the frequency of thyroid dysfunction and antibodies in 50 subjects with primary APS. Hypothyroidism was present in 22% of patients and thyroid autoantibodies in 18% of them. No clinical difference regarding thrombotic and obstetrical events was observed between APS patients with and without TAI. Mavragani et al. (27) tested 75 APS patients (40 primary APS and 35 APS secondary to SLE), 75 SLE patients and 75 healthy controls for anti-Tg and anti-TPO antibodies. No significant difference in the prevalence of thyroid antibodies was found between the three groups. However, SLE-APS patients (that is APS secondary to SLE) show significant increased rates of anti-TPO antibodies, but not primary APS patients, compared to healthy controls. More exciting is the fact that TAI identified a subgroup of APS patients with increased prevalence of ischemic central nervous system disease. Eighty-three percent of TAI-positive patients had evidence of central nervous system involvement vs 49% of TAI-negative patients. Authors speculated a cross-reactivity of these antibodies against shared epitopes between thyroid gland and central nervous system endothelium, such as α-enolase, an enzyme previously proposed to be involved in HT encephalopathy (30). Similarly, cross-reactivity between thyroid autoantibodies and CNS antigens had already been proposed by Le Donne et al. (31) to explain the neuropsychological perturbations observed in the postpartum. Innocencio et al. (26) were not able to replicate these results in a cohort of 63 patients including 25 RA, 25 SS, and 13 APS. None of the APS patients showed thyroid antibodies positive titers when compared with 13 and 8% of RA and SS subjects, respectively. In a large cohort of 203 women with primary obstetrical APS, De Carolis et al. (29) reported TAI in 27% of them. Of interest, as discussed later, patients with aPL antibodies alone had greater percentage of spontaneous pregnancies and live births when compared with patients positive for both thyroid antibodies and aPL antibodies.

On the basis of these data, it seems that the appearance of TAI in APS patients and conversely the occurrence of aPL in AITD patients is a fairly frequent phenomenon. The presence of aPL antibodies during thyroid disorders does not seem to have any clinical implication and more likely corresponds to hyperstimulation of self-reactive B-clones. However, their occurrence during an active GD, the latter representing by itself a risk factor for thrombosis, may in rare cases lead to thrombotic manifestations. But studies remain scarce and small, larger cohorts will be needed to confirm these findings. In addition, in most studies, especially in older ones, only ACL antibodies were measured. Moreover, some important data are lacking to correctly interpret the clinical significance of these antibodies, in particular the levels of aPL antibodies and the eventual positivity for the lupus anticoagulant test.

## APS, TAI, and Pregnancy

Although the association of aPL antibodies and antithyroid antibodies most often appears to have no clinical relevance, this may be of greater importance during pregnancy since both antibodies are recognized for their role in RM. RM is defined as three or more consecutive pregnancy losses with the same partner before 20 weeks of gestation (32). Etiologies include genetic, endocrine, anatomical, immunological, thrombophilic and environmental factors.

Antiphospholipid syndrome is the most important acquired risk factor for a treatable cause of recurrent pregnancy loss and can be found in 5–15% of cases (33). RM is part of APS clinical classification criteria (34, 35) that include vascular thrombosis and pregnancy morbidity. The latter criteria comprises: recurrent early miscarriage, late pregnancy loss, and prematurity due to placenta insufficiency or eclampsia/preeclampsia (35). Therefore, it is now widely accepted that aPL antibodies screening is an indispensable part of RM assessment. In addition, large randomized trials have allowed defining a standard of care based on antithrombotic treatment (36). Pathogenic mechanisms of pregnancy complications in APS are incompletely understood. aPL antibodies could reduce the proliferation and invasion of extra-villous trophoblasts, leading to placental mal-perfusion and finally to placental infarction, impaired spiral artery remodeling, decidual inflammation, increased syncytial knots and decreased vasculo-syncytial membranes (37).

Some endocrine disorders, such as diabetes mellitus, hyperprolactinemia and thyroid diseases have also been associated with miscarriage (38). The fact that overt hypothyroidism negatively affects pregnancy has been affirmed, but the implication of isolated TAI occurring in euthyroid women was still matter of debate. In 2011, Chen et al. (39) conducted a large meta-analysis including 22 studies: 14 cohort studies with 598 TAI-positive vs 4,870 TAI-negative pregnancies, and 8 case–control studies with 1,077 recurrent aborters. Overall, authors reported a significant higher risk of spontaneous miscarriage in euthyroid women with TAI (pooled OR = 2.55, *p* = 0.002 in case–control studies; pooled OR = 2.31, *p* < 0001 in cohort studies). The underlying mechanisms could consist of different aspects (39, 40): (A) heightened autoimmunity against foeto-placental unit, (B) direct involvement of thyroid autoantibodies interfering with trophoblast differentiation and proliferation, (C) induction of T-cell dysfunction causing an alteration of the endometrium that affects implantation, (D) higher age, and (E) mild hypothyroidism affecting reproductive outcome. Another hypothesis is that TAI may represent a marker for a global autoimmune state that is responsible for an elevated risk of reproductive failures rather than the actual cause of pregnancy losses. Anyway, as for aPL antibodies, the systematic search for TAI in women with RM is widely recommended (41).

Another important point to emphasize is that during pregnancy, GD may remain silent due to immune tolerance; But most often pregnancy and delivery can cause an onset and/or a flare-up of hyperthyroidism due to GD (42–44). The possible association with aPL antibodies is therefore important to consider since these antibodies may increase the thrombotic risk inherent in GD.

Consequently, the combination of these two major factors, namely, aPL antibodies and antithyroid antibodies, could be of great importance for better understanding and management of RM. Main studies are reported in Table 2. Recently, Promberger et al. (45) evaluated the association between aPL antibodies and antithyroid antibodies in a large cohort of 156 women with RM. Eighteen percent of women had either anti-TPO or anti-Tg positivity. In women with positive antithyroid antibodies, 13.8% had aPL antibodies compared to 2.4% in TAI-negative women. Moreover, women with both anti-TPO and anti-Tg antibodies exhibited higher titers of aPL antibodies. Interestingly, none of the parameters of autoimmunity was correlated with the number of previous pregnancy losses (45). Kim et al. (40) reported in 265 women with RM or unexplained infertility a 20% prevalence of TAI. They observed an increased prevalence of ACL antibodies in TAI-positive women compared with TAI-negative patients (27.8 vs 17.5%, *p* = 0.042). In addition, they found higher Th1/Th2 cytokines-expressing CD3+/CD4+ T cells ratios in women with TAI, suggesting the implication of Th1 immunity and pro-inflammatory status in the pathogenesis of TAI-related RM. Mecacci et al. (46) evaluated the prevalence of thyroid autoantibodies in 69 women with RM, fetal death or preeclampsia and investigated their association with other autoantibodies. Antithyroid antibodies were present in 37.9, 40.9, and 33.3%, respectively, of patients, which was significantly higher than in healthy controls (14.5%). Unlike previous studies, the prevalence of aPL antibodies was no significantly different in women positive (26.9%) and negative (34.9%) for TAI.

**Table 2:** APS, TAI, and pregnancy.

Study	Number of subjects	Population	Antibody	Outcome
De Carolis et al. (29)	203 patients 162 controls	Primary obstetrical APS patients	TgAb, TPOAb, aPL (type not specified)	Positive TAI in 27% of APS patients Reduced fertility and worst pregnancy outcome in TAI+ and TAI/APS women when compared with APS alone women
		Controls with TAI and RM		
Kim et al. (40)	256 patients	RM or unexplained infertility women	TgAb, TPOAb, aPL (6 types)	20% TAI positivity
				In TAI+ patients 27.8% ACL positivity vs 17.5% in TAI− patients
Promberger et al. (45)	156 patients	RM women	TgAb, TPOAb, ACL, β2GP1Ab	18% TAI positivity
				In TAI+ patients 13.8% aPL positivity vs 2.4% in TAI− patients
				No correlation with the number of miscarriages
Mecacci et al. (46)	69 patients	RM, fetal death, or preeclampsia women	TgAb, TPOAb, ACL, LA	TAI higher in patients (37.9, 40.9, and 33.3%) vs controls (14.5%)
	69 controls			No significant difference in aPL positivity

*ACL, anticardiolipin; aPL, antiphospholipid; β2GP1Ab, anti-β2GP1 antibodies; LA, lupus anticoagulant; RM, repeated miscarriage; TAI, thyroid autoimmunity; TgAb, antithyroglobulin antibody; TPOAb, antithyroperoxydase antibody; APS, antiphospholipid syndrome*.

But the most exciting trial to date on this topic was conducted by De Carolis et al. (29), to assess the presence of antithyroid antibodies in 203 women with primary obstetrical APS (aPL + RM) and compare APS alone with APS + TAI for fecundity and pregnancy outcome. It is to note that the type of aPL antibodies is not specified. A group of 162 women with TAI alone and RM served as controls. First, a 27% prevalence of TAI was found in APS subjects. Analyzing fecundity, 74% of APS-alone and 67% of total APS-positive women (APS alone and APS + TAI) became spontaneously pregnant, which was significantly higher than APS + TAI and TAI-alone women (48 and 49%, respectively). Therefore, women with TAI with or without APS had reduced fertility.

When pregnant, patients started a therapeutic regimen with high doses of intravenous immunoglobulin once a month until the 33rd week of pregnancy. Ninety-nine of the 136 pregnant women where thereafter followed. Pregnancy was successful for 92% of APS alone and 82% of total APS women, which once again was significantly higher than 60 and 57% in APS + TAI and TAI-alone women, respectively. In addition to reduced fecundity, TAI women had lower percentage of successful pregnancies. This study demonstrates the importance of TAI in patients with APS since it appears to be a stronger prognostic factor than aPL antibodies presence for fecundity and pregnancy outcome. Surprisingly, aPL and antithyroid antibodies do not seem to be synergic since rates are similar between TAI alone and TAI + APS groups. Authors conclude that TAI should always be evaluated in women with RM including those with aPL antibodies.

## Conclusion

In the light of these data, the association of TAI and aPL antibodies appears to be quite common. In most cases, this phenomenon has no clinical consequence and is likely the result of hyperstimulation of self-reactive B-clones. However, rarely, the combination of an uncontrolled GD which is recognized as a factor of hypercoagulability and aPL antibodies may promote the occurrence of thrombotic events. This situation should therefore lead to greater attention. In addition, one study interestingly highlighted a possible relationship between central nervous system involvement and TAI/APS association. Further studies will be required to investigate this hypothesis. Finally, special attention should be paid to women of childbearing age. Indeed, the presence of aPL antibodies, isolated or in the context of an autoimmune disorder, but even more so of TAI even in the absence of thyroid dysfunction are major and independent factors of reduced fertility and worse pregnancy outcome. Screening should therefore include systematically these two elements, and the monitoring and management of the pregnancy should be adapted.

## Author Contributions

MV conducted the review and wrote the article.

## Conflict of Interest Statement

The author declares that the research was conducted in the absence of any commercial or financial relationships that could be construed as a potential conflict of interest.

## Abbreviations

AITD, autoimmune thyroid diseases; aPL, antiphospholipid; APS, antiphospholipid syndrome; GD, Graves’ disease; HT, Hashimoto’s thyroiditis; SLE, systemic lupus erythematosus; TAI, thyroid autoimmunity; Tg, thyroglobulin; Th, T-helper; TPO, thyroperoxydase; TSHR, thyroid-stimulating hormone receptor.
